# Mental Health of Prostate Cancer Patients: Content Review on YouTube^TM^

**DOI:** 10.3390/ijerph20064721

**Published:** 2023-03-07

**Authors:** Benedetta Muzii, Francesco Di Bello, Fabio Carraturo, Tiziana Di Perna, Gianluigi Califano, Simone Morra, Francesco Mangiapia, Cristiano Scandurra, Luigi Giuliani, Giuseppe Celentano, Roberto La Rocca, Massimiliano Creta, Nicola Longo, Nelson Mauro Maldonato, Claudia Collà Ruvolo

**Affiliations:** 1 Department of Humanistic Studies, University of Naples Federico II, Via Porta di Massa 1, 80133 Napoli, Italy; 2 Department of Neuroscience, Reproductive Sciences and Dentistry, University of Naples Federico II, Via Sergio Pansini 5, 80131 Napoli, Italy; 3 Departmental Program of Clinical Psychopathology, Public Hospital of Naples Federico II, Via Sergio Pansini 5, 80131 Napoli, Italy

**Keywords:** prostate cancer, depression, YouTube, mental health, social media, urology

## Abstract

The aim of this study is to evaluate YouTube™ content in terms of the quality of information available about prostate cancer (PCa) in relation to incidence, symptomatology, and potential treatments for patients’ mental health. We searched on YouTube™ for terms related to mental health combined with those relating to prostate cancer. Tools for audio–visual-content PEMAT A/V, Global Quality Score, and DISCERN score were applied for the assessment of videos’ quality. A total of 67 videos were eligible. Most of the analyzed YouTube™ videos were created by physicians (52.2%) in contrast to other author categories (48.8%). According to the PEMAT A/V, the median score for Understandability was 72.7% and the overall median score for Actionability was 66.7%; the median DISCERN score was 47, which correspond to a fair quality. Only videos focusing on the topic “Psychological Effects and PCa treatment” were significantly more accurate. The General Quality Score revealed that the majority of YouTube™ videos were rated as “generally poor” (21, 31.3%) or “poor” (12, 17.9%). The results suggest that the content of YouTube™ videos is neither exhaustive nor reliable in the current state, illustrating a general underestimation of the mental health of prostate cancer patients. A multidisciplinary agreement to establish quality standards and improve communication about mental health care is needed.

## 1. Introduction

Prostate cancer (PCa) is one of the most common neoplasms among men, and its incidence rates are increasing worldwide [1,2]. A cancer diagnosis can have a negative impact on overall health [3,4]. In fact, the scientific literature highlights that a diagnosis of PCa may lead to a higher risk of developing mental disorders [5] and a higher prevalence of suicidal ideation [6,7]. This population may experience peculiar forms of psychological distress, such as a decrease in masculine self-esteem [8,9] and impaired body image [10,11] due to erectile dysfunction, impotence, and incontinence caused by surgery and/or pharmacological treatment, with a general prevalence of depressive and demoralization symptoms [7,8,9,10,11]. Indeed, PCa patients may benefit from exploiting their large social support networks, which, in turn, could be effective in reducing the risk of developing negative mental health conditions [12]. Quality of life and survivorship issues are critical for the all-round effective treatment of these patients, which may benefit psychological support in conjunction with conventional intervention programs [13,14]. Among the various coping strategies, PCa patients can approach their illness with health-information-seeking behaviors, which have the potential to deeply impact decisions and adjust processes to cope with illness [15]. Given that health information accessibility has exponentially increased due to search engines and social media [16,17,18], it is critical for health care professionals to be cognizant of the types of information that are available to patients and the general public [19,20]. In this context, YouTube™ is a social media website with a worldwide diverse communities, where a massive number of videos are uploaded daily [21,22,23]. The recent literature highlights that YouTube™ has great potential for outreach since it is a platform that is often pre-installed on a massive range of smartphones on the market, freely available, and captivating [24,25].

This type of media channel can also disseminate biased, scarce, or low-quality information about mental health with respect to PCa patients [26], thus hindering the prevention and recognition of psychological distress. An analysis of the available content that PCa patients may be exposed could provide health care workers with key information about the public understanding of mental health and the risk of self-stigma. Indeed, a recent YouTube™ content analysis on depression [27] showed that depression on YouTube™ is generally portrayed as chronic, recurrent, and mostly occurring due to biological or environmental factors, while psychological resources are rarely mentioned. Investigating how mental health awareness is portrayed on a vast and public platform such as YouTube may lead to the enrichment of health practices for PCa patients and the promotion of their psychological well-being throughout their lives [28].

To the best of our knowledge, no previous studies have specifically assessed mental health content shared on YouTube™ in relation to PCa. Thus, the aim of the current study is to assess the quality of the information available on YouTube™ about the prevalence, symptomatology, and potential treatments for mental health available in this specific population and whether this communication channel can be used as a reliable source.

## 2. Materials and Methods

### 2.1. Search Strategy

Following PRISMA guidelines [29], we performed a systematic research on YouTube.com on 18 May 2022 at 9.50 a.m. (CEST). The research was executed while logged out from any personal account and in the browser’s incognito mode. The videos were searched using YouTube^TM^’s default search setting, which is the “Relevance” filter. The search string was composed of terms related to prostate cancer (e.g., “Prostate cancer”, “Prostate tumor”, and “Prostate neoplasm”) combined with mental health keywords (e.g., “Depression”, “Anxiety”, and “Distress”), amounting to a total of 36 different keyword combinations (see Supplementary Material). For each combination, we recorded the first 30 videos that arose in the search; this cutoff was selected to include videos that were most likely to be viewed based on typical internet-scrolling behavior [17]. Since one combination yielded the result of 31 videos, we decided to include the leftover video in the study.

### 2.2. Selection Criteria and Procedure

**Figure 1:**
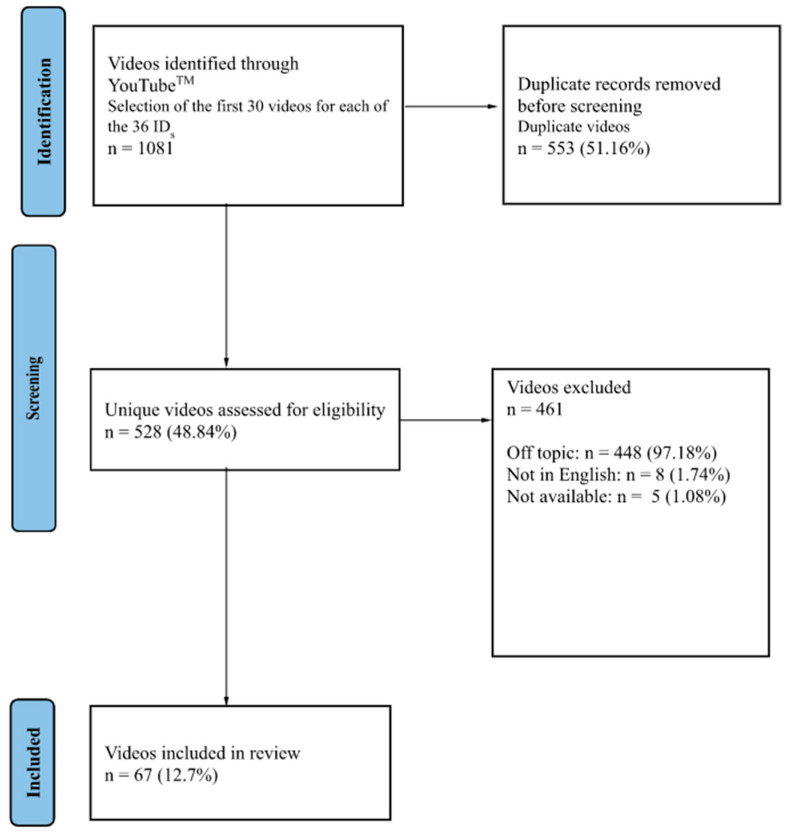
Prisma flow chart.

A total of 1081 videos were collected and screened in the next few days through the following inclusion criteria: (1) videos had to reference mental health and (2) be in English. After the exclusion of duplicate videos (*n* = 553, 51.16%), 528 videos were assessed for eligibility. The following exclusion criteria were applied: off-topic videos (*n* = 448, 97.18%), non-English language videos (*n* = 8, 1.74%), and video not available (*n* = 5, 1.08%). A total of 67 (12.7%) videos were eligible for the analyses (Figure 1). Therefore, the following variables were recorded on 20 May 2022: the video length (in seconds), views, persistence time on YouTube^TM^ (in days), likes, comments, subscribers, view ratio (defined as the ratio between the number of views and the persistence time on YouTube^TM^), and number of videos with disabled comments. Furthermore, we collected additional information on (a) target audience; (b) authoring institution; (c) topic; and (d) year of upload (<2014 vs. 2015–2017 vs. 2018–2019 vs. 2020–2022). Specifically, target audience was categorized as Patients or Healthcare Workers. Authoring institution category included hospitals, private users, medical doctors, news agencies, nonprofit organizations and scientific societies. Finally, the topics were categorized as follows: “Psychological effects (PEs) of PCa and its diagnosis, Psychological effects of PCa treatments, patient’s experience of his illness and its management, and information about tools for taking care of PCa PEs.”

### 2.3. Strategies and Tools for Video Content Assessment

Video content was independently assessed by a psychiatrist (LG) and two psychologists in training (FC and TDP), all of whom were staff members of the “Departmental Program of Clinical Psychopathology” of the University of Naples “Federico II”. Two additional researchers, a licensed psychologist from the same department and a urologist, clarified discrepancies; subsequently, a consensus was reached among all reviewers. Each video was evaluated through the following standardized tools.

#### 2.3.1. Patient Education Materials Assessment Tool for Audiovisual Materials

To assess and compare the understandability and actionability of educative content, we used the Patient Education Materials Assessment Tool for Audiovisual Content (PEMAT A/V) [30]. This tool contains 17 items addressing understandability (items 1–13) and actionability (items 14–17), with three possible answers (agree = 1, disagree = 0, and not available = NA). The total score was then calculated through three main steps. First, only the total points for the material on the Understandability items were summed. Second, the sum was divided by the number of items for which the material was rated (the items that were scored as NA were excluded). Finally, the result was multiplied by 100. The same process was performed for Actionability items. At the end, the results were expressed as a percentage, where higher scores indicate more understandable and/or actionable content.

#### 2.3.2. DISCERN

DISCERN [31] is a standardized questionnaire designed to judge the quality of information regarding treatment choices for a health problem. It consists of 16 items aiming to assess the reliability of the material, the quality of the information regarding the treatment choices, and the overall quality of the publication as a source of information. The frequency for each item was measured on a 5-point Likert scale ranging from 1 (‘Poor quality’) to 5 (‘High quality’) with the following total score categories: ‘excellent’ (range 63–80), ‘good’ (range 51–62), ‘fair’ (range 39–50), ‘poor’ (range 27–38), and ‘very poor’ (range 16–26).

#### 2.3.3. Global Quality Score

The Global Quality Score (GQS) is a tool for assessing the quality, feasibility, and clinical utility of a video [32]. Five possible scores from 1 (poor quality, poor flow, most of the information is missing, and not at all useful) to 5 (excellent quality, excellent flow, completely accurate information, and very useful) were assigned.

### 2.4. Statistical Analyses

Descriptive statistics were presented as medians and interquartile ranges (IQR) for continuously coded variables or counts and percentages for categorically coded variables. Chi-square distribution was used to test the statistical significance of proportions’ differences. The ANOVA and the Kruskal–Wallis tests were used to examine the statistical significance of medians’, means’, and distributions’ differences. In all statistical analyses, R software (www.rproject.org) environment for statistical computing and graphics (R version 4.0.0) and Microsoft Excel 2019 were used. All tests were 2-sided, with a level of significance set at *p* < 0.05.

## 3. Results

### 3.1. Videographic Characteristics

Of all the 1081 videos collected, 67 were deemed suitable for the analyses (Table 1). The mean length and views recorded were 1358.9 (Standard deviation [SD]: 201.0) and 4277.3 (SD: 1388.5), respectively. The mean value of the view ratio was 2.7 (SD: 1.3; Range 0–83). The number of YouTube™ videos produced by medical doctors, hospitals, private users, and others was 35 (52.2%), 10 (14.9%), 13 (19.4%), and 9 (13.4%), respectively. According to the uploading years (<2014 vs. 2015–2017 vs. 2018–2019 vs. 2020–2022), 16 (23.9%) vs. 17 (25.4%) vs. 17 (25.4%) vs. 22 (32.8%) videos were uploaded on YouTube™, respectively. The videos were initially stratified according to the target audience: 14 (20.9%) were addressed to healthcare workers vs. 53 (79.1%) addressed to patients. Then, the YouTube™ videos were stratified according to their topics: 17 (25.4%) referred to patient experience, 17 (25.4%) referred to PEs and PCa treatment, 22 (32.8%) to PEs and PCa diagnosis, and 11 (16.4%) to tools for managing PCa PEs.

### 3.2. Video Quality Assessment

According to PEMAT A/V (Table 2), the overall mean Understandability score was 72.3 (SD: 1.9) and the overall mean Actionability score was 58.6 (SD: 3.8). Specifically, according to the target audience (healthcare workers vs. patients), the mean Understandability score was 64.5% (SD: 4.1) vs. 74.4% (SD: 2.1), respectively (*p* = 0.04), and the mean Actionability score was 52.4 (SD: 7.5) vs. 60.2 (SD: 4.4), respectively (*p* = 0.3). No statistically significant differences were recorded concerning the videos’ topics (*p* > 0.05).

According to GQS (Table 3), 21 (31.3%) of the eligible YouTube™ videos showed a “generally poor” quality, 12 (17.9%) were “poor”, 16 (23.9%) were “medium”, 15 (22.4%) were “good”, and only 3 (4.5%) reached “excellent” evaluation. According to the video topic, 58.8% of the videos referring to patients’ experience were “generally poor” (*p* = 0.04). No additional statistical differences were recorded in the GQS analysis.

According to the DISCERN tool (Table 4), only 36 videos mentioning treatment choices were analyzed. The overall mean DISCERN score was 48.1 (SD: 2.4). According to the target audience (healthcare workers vs. patients), the mean DISCERN scores were 50.6 (5.3) vs. 47.4 (SD: 2.8), respectively (*p* = 0.6). According to the topic (Patient Experience, PEs and PCa treatment, PEs and PCa diagnosis, and tools for managing PCa PEs), the mean DISCERN scores were 41.5 (SD: 4.0) vs. 47.8 (SD: 3.6) vs. 55.9 (SD: 3.5) vs. 41.2 (SD: 7.9), respectively (*p* = 0.02). Specifically, the mean DISCERN scores of SECTION 1 were 20.8 (SD: 1.5) vs. 27.5 (SD: 1.5) vs. 31.1 (SD:1.5) vs. 22.4 (SD:3.5), respectively (*p* = 0.01).

### 3.3. Video Quality Assessment over Time

**Figure 2:**
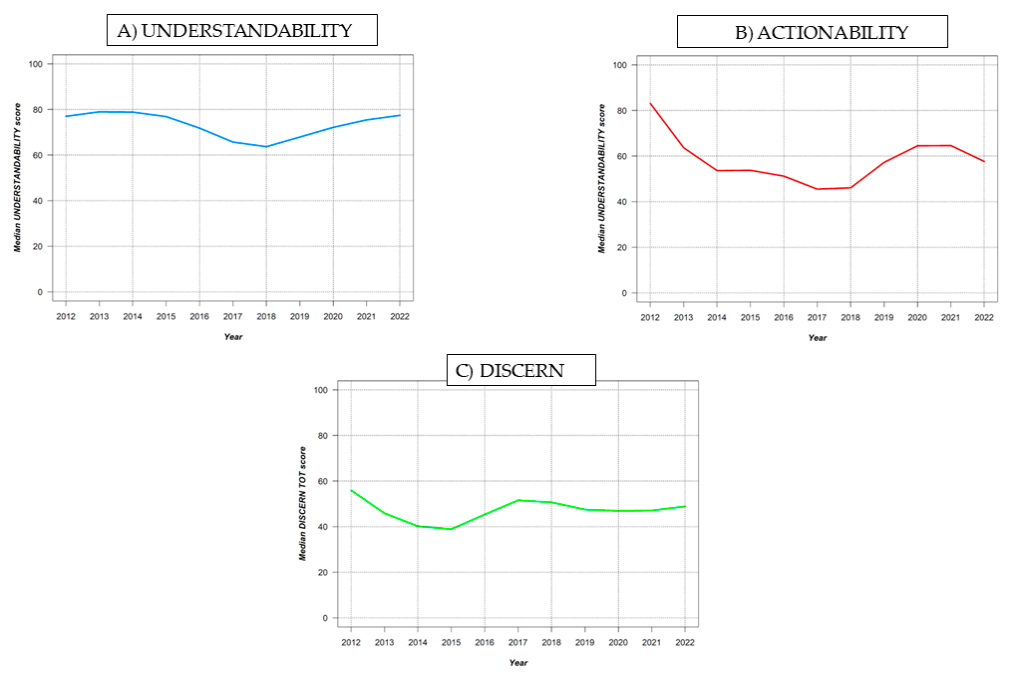
Understandability, Actionability, and DISCERN total score trends over time.

According to the median Understandability (*p* = 0.9), Actionability (*p* = 0.1), and DISCERN total (*p* = 0.2) scores, no statistically significant difference was recorded over time (2012–2022) (Figure 2).

## 4. Discussion

The aim of the current study was to evaluate the quality of YouTube^TM^ videos on the mental health of PCa patients and to investigate the reliability of the information currently available. Several interesting observations can be derived from our data analysis. Throughout the interpretation of the samples’ videographic characteristics, it was found that the videos reviewed had a median length of around 5 min, but had a very low number of views, likes, and comments, resulting in a low view ratio. This result differs from a recent YouTube™ content analysis on PCa: according to Basch et al. [19], the most viewed videos in 2016 had views per video that ranged between 34,004 and 13,312,255, with thousands of views per day. In addition, it was found that all the videos focusing on providing general information about PCa omitted mental health aspects. This observation may suggest that the videos mentioning mental health are not among the most engaging content concerning PCa. Of the 67 eligible videos, most are aimed at PCa patients, but the quality of their educational information is low: according to PEMAT A/V, the overall mean Understandability score was 72.3% while the overall mean Actionability score was 58.6%; for both scores, audio–visual content can be defined as understandable and/or actionable if the score is higher than 70% [30]. Moreover, videos dealing with patients’ experience have lower levels of Actionability and Understandability compared to the videos focusing on more scientific content; indeed, GQS revealed a generally low score, with 94.1% of the eligible videos falling in the Medium to Poor quality range. The sharing of cancer experiences can be ambiguous due to the subjective nature of how events are processed. Indeed, patients who have already undergone surgery and/or pharmacological treatments share their emotional experiences, which may foster a sense of hope and compassion for other patients with early-stage PCa [33,34], although this may not be sufficient to enhance awareness of the disease’s mental health risks, symptoms, and feasible treatments. In addition, the videos aimed at patients have greater Understandability compared to content addressed to Healthcare Workers, but there are no major differences in GQS. The data analysis that was performed in accordance with DISCERN resulted in a medium score of 48.1, indicating a fair quality of the videos’ accuracy regarding treatment options and thus highlighting the need to improve the dissemination of reliable information on this specific topic. Only the videos on “PEs and PCa treatment” differed significantly from the other topics, with a mean of 55.9 (*p* < 0.05), which corresponds to a good evaluation. However, the analysis revealed no relevant differences between the videos dedicated to patients and the content addressed to healthcare workers; therefore, it is plausible that videos on mental health uploaded on YouTube™ are not a useful source of educational content for both the public and healthcare professionals. Furthermore, through an evaluation of the overall scores split by the years in which the videos were uploaded (Figure 2), we observed that following the SARS-CoV-2 pandemic, both the number of videos and the quality or their content were unvaried, even though the global prevention measures adopted to contain the infection had fostered telemedicinal practices to support, educate, and address patients’ needs [35,36].

## 5. Conclusions and Limitations

Notwithstanding the fact that our methodology followed standardized research criteria shared in the scientific literature on content analysis of YouTube^TM^ videos [37,38,39], the current study has some limitations. Primarily, YouTube™ search results are influenced by Google’s own search algorithms, which are supported by users’ previous search activities, but the methodological decision to undertake the search in incognito mode likely reduced this bias. Second, only English-language videos were included in this study, but content in other languages could provide different information and highlight cultural imbalances. The keywords selected could entail a selection bias, and some reliable or unreliable content can be excluded during data collection. To prevent this risk, we used 36 keyword combinations and collected as many videos as possible. In addition, YouTube^TM^ is a multimedia platform in unrestrained growth, and its content can change rapidly over time with new updates, so our study is limited to a certain period of time and the analyzed data may quickly be altered. In summary, the overall content of YouTube^TM^ videos about the incidence, prevention, and therapeutic options related to the mental health of PCa patients is not comprehensive and reliable in its current state, with the exception of a few videos that focus on the psychological side effects of a medical PCa regimen. These findings indicate that mental healthcare’s effectiveness in improving cancer patients’ quality of life [40] is substantially underestimated. The same observation can be made for participation in preventive and early diagnosis awareness campaigns [41]. Future research may focus on different social networks and media channels so that scientific societies and healthcare systems may benefit from this knowledge to promote the broad circulation of trustworthy information for patients and to educate HCWs. Thus, these results suggest that a multidisciplinary agreement is needed to define high-quality standards and improve communication in order to provide essential information for mental health care awareness after a PCa diagnosis and, therefore, support a key factor of adherence to medical treatments and the maintenance of a good quality of life.

## Figures and Tables

**Table 1:** Videographic characteristics of 67 YouTube^TM^ videos on mental health of PCa patients selected on 18 May 2022.

Videographic Characteristics		Overall *n* = 67
Length, s	Mean (SD)	1358.9 (201.0)
	Median (IQR)	332 (191.5–2550.5)
	Min–Max	52–6035
Views, *n*	Mean (SD)	4277.3 (1388.5)
	Median (IQR)	444 (131–1597)
	Min–Max	3–74,650
Persistence time on YouTube, days	Mean (SD)	1773.1 (150.2)
	Median (IQR)	1597 (748–2546)
	Min–Max	74–5183
Likes, *n*	Mean (SD)	29.1 (9.0)
	Median (IQR)	3 (1–15)
	Min–Max	0–495
Comments, *n*	Mean (SD)	2 (0.6)
	Median (IQR)	0 (0–2)
	Min–Max	0–23,000
Subscribers, *n*	Mean (SD)	57,958.7 (32,912.9)
	Median (IQR)	4440 (1060–16,800)
	Min–Max	0–1,860,000
View ratio	Mean (SD)	2.7 (1.3)
	Median (IQR)	0 (0–0)
	Min–Max	0–83
Disabled comments, *n* (%)	Yes	10 (14.9)
	No	57 (85.1)
Target audience, *n* (%)	Healthcare workers	14 (20.9)
	Patients	53 (79.1)
Authoring institution, *n* (%)	Hospital	10 (14.9)
	Private user	13 (19.4)
	Medical doctor	35 (52.2)
	Other	9 (13.4)
Topic, *n* (%)	Tools for managing PCa’s psychological effects	11 (16.4)
	Psychological effects and PCa treatment	17 (25.4)
	Patient experience	17 (25.4)
	Psychological effects and PCa diagnosis	22 (32.8)
Year, range, *n* (%)	<2014	16 (23.9)
	2015–2017	17 (25.4)
	2018–2019	13 (19.4)
	2020–2022	21 (31.3)

Abbreviation: s = seconds; n = number; IQR = interquartile range; Pca = Prostate cancer; SD: standard deviation.

**Table 2:** PEMAT audio/visual (A/V) Understandability and Actionability of 67 YouTube videos on mental health of Pca patients stratified according to target audience (healthcare workers vs. patients) and topic (patient experience vs. psychological effects and PCa diagnosis vs. psychological effects and PCa treatment vs. tools for managing PCa psychological effects).

			Target Audience			Topic				
		Overall *n* = 67	HCWs *n* = 14 (20.9%)	Patients *n* = 53 (79.1%)		Patient experience *n* = 17 (25.3%)	PEs and PCa diagnosis *n* = 22 (32.8%)	PEs and PCa treatment *n* = 17 (25.3%)	Tools for managing PCa PEs *n* = 11 (16.4%)	
**PEMAT A/V**					*p*					*p*
Understandability	Mean (SD)	72.3 (1.9)	64.5 (4.1)	74.4 (2.1)	0.04	64.4 (3.9)	77.2 (2.7)	72.8 (4.4)	74.2 (3.5)	0.06
	Mdn(IQR)	72.7 (64.6–82.6)	66.7 (55.6–69.8)	77.8 (66.7–83.3)	0.02	62.5 (55.6–77.8)	76.4 (66.7–83)	72.7 (55.6–90)	70 (66.7–81.2)	0.06
Actionability	Mean (SD)	58.6 (3.8)	52.4 (7.5)	60.2 (4.4)	0.3	41.2 (7.3)	66.7 (5.8)	62.7 (7.4)	62.9 (10.7)	0.06
	Mdn(IQR)	66.7 (33.3–66.7)	50 (33.3–66.7)	66.7 (33.3–100)	0.3	33.3 (33.3–66.7)	66.7 (66.7–91.7)	66.7 (33.3–100)	66.7 (33.3–100)	0.06

Abbreviation: *p* = *p*-value; HCWs = healthcare workers; Mdn = Median; IQR = interquartile range; PCa = prostate cancer; Pes = psychological effects; SD: standard deviation.

**Table 3:** Global Quality Score values of 67 YouTube videos on mental health of PCa patients stratified according to target audience (healthcare workers vs. patients) and topic (patient experience vs. psychological effects and PCa diagnosis vs. psychological effects and PCa treatment vs. tools for managing PCa psychological effects).

		Target Audience			Topic				
	Overall *n* = 67	HCWs *n* = 14 (20.9%)	Patients *n* = 53 (79.1%)		Patient experience *n* = 17 (25.3%)	Pes and PCa diagnosis *n* = 22 (32.8%)	Pes and PCa treatment *n* = 17 (25.3%)	Tools for managing PCa Pes *n* = 11 (16.4%)	
**Global Quality Score**	*n* (%)	*n* (%)	*n* (%)	*p*	*n* (%)	*n* (%)	*n* (%)	*n* (%)	*p*
Excellent	3 (4.5)	1 (7.1)	2 (3.8)	0.9	0 (0)	0 (0)	2 (11.8)	1 (9.1)	0.2
Good	15 (22.4)	2 (14.3)	13 (24.5)	0.6	1 (5.9)	7 (31.8)	6 (35.3)	1 (9.1)	0.1
Medium	16 (23.9)	3 (21.4)	13 (24.5)	0.9	2 (11.8)	9 (40.9)	3 (17.6)	2 (18.2)	0.1
Generally poor	21 (31.3)	5 (35.7)	16 (30.2)	0.9	10 (58.8)	4 (18.2)	4 (23.5)	3 (27.3)	0.04
Poor	12 (17.9)	3 (21.4)	9 (17)	0.9	4 (23.5)	2 (9.1)	2 (11.8)	4 (36.4)	0.2

Abbreviation: *p* = *p*-value; HCWs = healthcare workers; PCa = prostate cancer; Pes = psychological effects.

**Table 4:** DISCERN instrument Section 1, Section 2, and Section 3 of 67 YouTube videos on mental health of PCa patients stratified according to target audience (healthcare workers vs. patients) and topic (patient experience vs. psychological effects and PCa diagnosis vs. psychological effects and PCa treatment vs. tools for managing PCa psychological effects).

			Target Audience			Topic				
		Overall *n* = 36	HCWs *n* = 8 (22.2%)	Patients *n* = 28 77.8%)		Patient experience *n* = 4 (11.1%)	PEs and PCa diagnosis *n* = 13 (36.1%)	PEs and PCa treatment *n* = 11 (30.6%)	Tools for managing PCa PEs *n* = 8 (22.2%)	
**DISCERN**					*p*					*p*
Section 1: Is the publication reliable? ^a^	Mean (SD)	26.7 (1.2)	30.6 (2.2)	25.6 (1.3)	0.07	20.8 (1.5)	27.5 (1.5)	31.1 (1.5)	22.4 (3.5)	0.01
	Mdn(IQR)	26.5 (22–32.2)	32.5 (24.5–35.2)	26 (20–30)	0.1	21 (19.2–22.5)	27 (25–30)	33 (27–35.5)	21.5 (16.2–28.5)	0.01
Section 2: How good is the quality of information on treatment choices? ^b^	Mean (SD)	18.5 (1.2)	17.2 (3.3)	18.8 (1.3)	0.6	18.2 (2.5)	17.5 (1.9)	21.2 (2.5)	16.5 (3.1)	0.06
	Mdn (IQR)	17.5 (12–23.2)	14.5 (11.2–21.5)	18.5 (12–23.2)	0.5	18.5 (15.8–21)	15 (12–23)	20 (16–28.5)	15 (10.8–17.8)	0.06
Section 3: Overall Quality rating ^c^	Mean (SD)	2.9 (0.2)	2.8 (0.5)	3 (0.2)	0.6	2.5 (0.5)	2.8 (0.4)	3.6 (0.3)	2.4 (0.5)	0.2
	Mdn(IQR)	3 (2–4)	3 (1.8–3.2)	3 (2–4)	0.6	3 (2.5–3)	3 (2–4)	3 (3–4.5)	2 (1–3.2)	0.2
DISCERN score	Mean (SD)	48.1 (2.4)	50.6 (5.3)	47.4 (2.8)	0.6	41.5 (4.0)	47.8 (3.6)	55.9 (3.5)	41.2 (7.0)	0.02
	Mdn(IQR)	47 (37–55.2)	49 (37.8–57)	47 (36.8–55.2)	0.7	43.5 (39.8–45.2)	43 (7–55)	53 (47–66)	38.5 (27.5–50.5)	0.02

Notes. ^a^ Sum Section 1 (max score 40), Questions 1–8; ^b^ Sum Section 2 (max score 35), Questions 9–15; ^c^ “Based on the answers to all the above questions, rate the overall quality of the publication as a source of information about treatment choices” (max score 5). DISCERN score has been categorized as follows: “excellent” (range 63–80), “good” (range 51–62), “fair” (range 39–50), “poor” (range 27–38), and “very poor” (range 16–26). Abbreviation: *p* = *p*-value; HCWs = healthcare workers; PCa = prostate cancer; Pes = psychological effects, SD: standard deviation.

## Data Availability

The data presented in this study are openly available.

## Supplementary Materials

The following supporting information can be downloaded at: https://www.mdpi.com/article/10.3390/ijerph20064721/s1, File S1: The search string composed of terms related to prostate cancer combined with mental health keywords has been reported in Supplementary Materials.
