# Supplementary material for: Mental Health of Prostate Cancer Patients: Content Review on YouTubeTM
Source: Int J Environ Res Public Health. 2023 Mar 7;20(6):4721. doi: 10.3390/ijerph20064721 (PMC10048673; doi:10.3390/ijerph20064721)
Supplement: Supplementary file 1 [file ijerph-20-04721-s001.zip › ijerph-2224504-supplementary.pdf]

Supplementary File S1

Search keyword combinations used on YouTube™ on the 18 May 2022.

Search keyword combinations

1. Prostate cancer AND Depression
2. Prostate cancer AND Mental Health
3. Prostate cancer AND Anxiety
4. Prostate cancer AND Stress
5. Prostate cancer AND Distress
6. Prostate cancer AND Mood
7. Prostate cancer AND Psychological effects
8. Prostate cancer AND Psychological well-being
9. Prostate cancer AND Quality of life
10. Prostate adenocarcinome AND Depression
11. Prostate adenocarcinome AND Mental Health
12. Prostate adenocarcinome AND Anxiety
13. Prostate adenocarcinome AND Stress
14. Prostate adenocarcinome AND Distress
15. Prostate adenocarcinome AND Mood
16. Prostate adenocarcinome AND Psychological effects
17. Prostate adenocarcinome AND Psychological well-being
18. Prostate adenocarcinome AND Quality of life
19. Prostate neoplasm AND Depression
20. Prostate neoplasm AND Mental Health
21. Prostate neoplasm AND Anxiety
22. Prostate neoplasm AND Stress
23. Prostate neoplasm AND Distress
24. Prostate neoplasm AND Mood
25. Prostate neoplasm AND Psychological effects
26. Prostate neoplasm AND Psychological well-being
27. Prostate neoplasm AND Quality of life
28. Prostate tumor AND Depression
29. Prostate tumor AND Mental Health
30. Prostate tumor AND Anxiety
31. Prostate tumor AND Stress
32. Prostate tumor AND Distress
33. Prostate tumor AND Mood
34. Prostate tumor AND Psychological effects
35. Prostate tumor AND Psychological well-being
36. Prostate tumor AND Quality of life
